# Composite Materials with Epoxy Resin Matrix and Natural Material Reinforcement—Pine Chips and Basalt Particles—Abrasive Properties Determination

**DOI:** 10.3390/ma18174038

**Published:** 2025-08-28

**Authors:** Robert Polasik, Sandra Kruszyńska, Aleksander Kwiatkowski

**Affiliations:** Faculty of Mechanical Engineering, PBS University of Science and Technology, Kaliskiego 7 Street, 85-789 Bydgoszcz, Poland or sbrauer09@gmail.com (S.K.); or aleksander.kwiatkowski00@gmail.com (A.K.)

**Keywords:** composite materials, green composites, geopolymer composites, abrasive properties, wood–resin composites, basalt–resin composites, mechanical properties, sustainable materials manufacturing

## Abstract

The article presents the results of original research on determining the abrasive properties of composite materials with an epoxy resin matrix reinforced with basalt particles in the form of powder and pine chips from the post-production waste of wooden elements. There are many studies available in the literature on the modification of composite materials in terms of achieving the required strength properties, but there is little information available in the area of achieving specific functional properties of composite materials, e.g., abrasive properties. Three composite materials with different proportions of the material components were made. These materials were tested using standardized tests to determine their mechanical properties, and these properties were compared in relation to the matrix material (epoxy resin). In order to determine the abrasive properties, an original research stand was made, on which the composites were tested using counter-samples made of an aluminum alloy. The mass loss of samples and counter-samples after the friction test was measured and determined. Changes in the electrospindle supply current and rotational measurements were also made. The values measured and determined in the tests were used as indicators of the abrasiveness of composite materials. It was shown that both the loss of mass of the sample and counter-sample and the parameters of the electrospindle operation are good, convenient indicators of the abrasive properties of the tested materials. The obtained results were subjected to statistical analyses. Optical 3D scans of the surfaces of exemplary samples were presented.

## 1. Introduction

Composites based on reinforcement from natural materials have attracted wide interest in the last few decades, especially due to their low cost, ease of production and low weight-to-volume ratio. Their biodegradability and recyclability are also of great importance, which is important due to the increasing public awareness of environmental problems. The need to improve previously known solutions and the constant search for new ones have accompanied man since the dawn of civilization. It is no different in the field of materials, including composites. Over the centuries, there has been a significant development of composites—from bricks made of mud and straw, through arches made of wood, bone and silk, to modern composites reinforced with glass or carbon fibers [1]. Along with the progress in the field of composites, the ecological awareness of their users and knowledge about recycling have also increased. Society is increasingly looking for solutions based on environmentally friendly products. Green composites, which combine beneficial features with an ecological aspect, have gained particular importance [2]. In addition to their pro-environmental character, natural composites are also more affordable. Often, reinforcements may be by-products or waste of other processes or materials. Polymer matrix composites (PMCs) are manufactured using thermoplastic and thermosetting resins. Examples of these groups include polyesters, epoxy resins, phenolic resins, silicones, furans and polyamides. Polymer composites are characterized by a relatively low weight compared to other composite materials. Additionally, thanks to their ease of mechanical processing, they can be given a form and properties that are closely matched to specific applications. Glass and carbon fibers are most often used as reinforcement. The main reason for why polymer matrix composites are chosen for the production of components is the weight savings in relation to their stiffness and strength [3]. The chosen reinforcement material has a significant impact on the final mechanical and functional properties of the resulting material. It is responsible for the tensile, compressive, and bending strength of the composite material [4].

### 1.1. Composites with Natural Fiber Reinforcement Materials

In article [5], the epoxy composites reinforced with *Lantana camara* fibers were described. The ability of the composite to absorb moisture and its effect on mechanical properties were investigated. Samples of composites reinforced with different weight percentages of fibers were prepared by a hand lay-up technique. An increase in tension and flexural strength was observed with increasing fiber content up to 30%. In the description of research work [6], a natural material, banana fibers, was used. To improve mechanical properties, banana fibers were hybridized with sisal fibers. Banana fiber/epoxy composite material tensile strength, bending strength, impact strength and water absorption were tested. The studies showed that the addition of sisal fibers to epoxy composites with banana fibers in an amount of up to 50 wt.% increased mechanical properties and reduced the ability to absorb moisture. In study [7], epoxy composites reinforced with recycled cellulose fibers (RCFs) with a fiber content of 19, 28, 40 and 46 wt.% in paper form were pre-dried at 70 °C, completely immersed in epoxy resin and then pressed at a pressure of 8.2 kPa. The results showed that the flexural strength, modulus of elasticity, fracture toughness and impact strength increased with increasing fiber content. The best mechanical properties were obtained with a fiber content of 46 wt.%. The authors of study [8] considered the possibility of using natural silk fibers as an alternative to glass fibers. Composites in the form of laminates were obtained using RTM technology (resin transfer molding). During the tests, it was found that the specific mechanical properties of silk/epoxy laminates are anisotropic and can be compared to the properties of glass fiber/epoxy laminates. Typically, silk composites showed an improvement in specific bending strength along the main direction of the weave in relation to the glass fiber/epoxy laminate by up to 23%. The tests also examined the effect of fabric moisture on the properties of the obtained composite. Paper [9] describes the research on composites reinforced with natural fibers such as *Prosopis juliflora*, Abutilon indicum and *Tapsi*. These are fibers originating from the Nallamalla forest region in India. The tested composites were manufactured in the form of laminates using the hand-laying method. The mass content of fibers ranged from 10 to 25% by weight. Among the tested samples, the samples with *Prosopis juliflora* fibers showed better mechanical properties and wear resistance compared to the other two fibers. The abrasion resistance of all three fibers was significantly improved compared to pure epoxy. Publication [10] concerns the use of pine fibers, in the form of pine needles, in an epoxy composite. Before use, the needles were dried in a laboratory oven to remove moisture. During the tests, it was found that mechanical properties such as tensile strength, bending strength and impact strength depend to a large extent on the content of fibers in the composite material. With the increase in the percentage of natural material, the impact strength decreased significantly to 53 MPa, while the tensile strength increased for 10% of the pine needle content and then decreased significantly with the increase in the needle content. A similar trend was observed for tensile strength. Mechanical properties of flax-fiber-reinforced epoxy composites and a comparison of the properties depending on the chemical treatment of the fiber were presented and discussed in paper [11]. The flax-fiber-reinforced epoxy composites were prepared by the autoclave molding method. The influence of various fiber parameters, such as lignin content, pectin content and degree of polymerization, on the composite properties was studied. In order to improve the fiber–matrix interactions, fiber surface modifications were performed, such as alkali treatment, silane treatment, isocyanate treatment and others. In most cases, an improvement in strength properties was observed. The widely described topic of the influence of chemical treatment on mechanical properties is important due to the wide application of this treatment in numerous studies over the years. Flax fibers have been studied many times over the years [12,13,14]. In 2014, researchers from India conducted experimental studies to compare the mechanical properties of jute-fiber-reinforced composites using epoxy and polyester resin matrices. The jute fibers were chemically treated with 5% and 10% NaOH solutions. The tensile strength of the jute–epoxy and jute–polyester composites was found to be 12.46 MPa and 9.23 MPa (for 5% NaOH). Composites with fibers treated with 5% NaOH also had higher tensile strength than those treated with 10% NaOH (by 18.67% for the epoxy composite and by 16.67% for the polyester composite). The flexural strength of jute–epoxy and jute–polyester composites was estimated at 38.68 and 44.71 MPa, respectively [15].

### 1.2. Composites with Non-Fibrous Wooden and Geological Origin Reinforcement Materials

In publication [16], Sundi wood dust was used as reinforcement in the epoxy composite. Composite samples were produced with seven different filler weight contents (from 0 to 10 wt.%). Tensile and bending tests were performed at three different speeds to investigate the mechanical behavior of the composites. The observations show that the mechanical properties increase up to a certain filler weight content and then gradually decrease. The best mechanical properties were observed for 10 wt.% filler and tensile test speeds of 1 mm/min and 2 mm/min. Scientific report [17] concerns composites with reinforcement from oak wood waste and peanut shells in relation to dispersion in epoxy resin. All composites with the addition of lignocellulose were rated as having a higher compressive strength (from 63.2 to 69.1 MPa). Samples containing wood flour as a filler exhibited better mechanical properties compared to samples with peanut shell flour. The tensile strength was 53.53 MPa and 42.74 MPa for the compositions containing 5 wt.% wood and peanut shell flour, respectively, after silanization. In work [18], the influence of the reinforcement of epoxy composites with pine sawdust depending on its weight content on their strength was described. The obtained results showed that composites with 15% of sawdust by weight content were determined as having the highest tensile strength, 7.5 MPa, and bending strength, 8.9 MPa, in comparison with composites with 5 wt.%, 30 wt.%, 40 wt.% and 50 wt.%. Moreover, the ability to absorb water was checked depending on the weight percentages. It was found that, with the introduction of a larger amount of sawdust, the water absorption by the composite increased. Basalt is commonly used in construction, especially in road construction as an aggregate added to concrete. This is related to its properties, such as compressive strength, abrasion resistance, frost resistance, and low water absorption, and it is also non-flammable and chemically neutral. It is widely used in organic farming, especially where the soil is significantly impoverished and barren. It is usually not a stand-alone fertilizer but rather a supplement to organic fertilizers such as compost, biohumus or manure. The use of basalt (in the form of flour) as one of the components of composites has so far been marginalized in the world of science. Another basalt product, i.e., basalt fibers, is very popular among researchers. This topic has been extensively described in the literature [19,20,21]. The use of basalt flour in composite materials was described in the area of the effect of its addition, as a replacement for quartz sand, to fly-ash-based geopolymers. It was found that basalt flour significantly increases compressive strength—by about 106%—and additionally causes an increase in bending strength by 11%. There was also a decrease in porosity in the structure of the tested geopolymers [22]. The results obtained in the above studies show a beneficial improvement in the properties of composites using basalt flour and encourage the extension of research in other material areas.

A literature review of the use of basalt flour and wood chips (production waste) indicates a lack of research on composite materials containing such reinforcements. Commonly used natural reinforcement materials are basalt fibers and wood dust. Wood chips can be a desirable/good reinforcement material because they combine the characteristics of fibrous materials used to make laminates and materials used in the dispersed phase (e.g., dust); they possess favorable mechanical properties and are easy to apply. Furthermore, they are readily available and affordable, as they are generally waste materials. Composites made from natural materials, such as basalt flour, pine chips and polyester resin, especially those made of recycled materials [23], are environmentally friendly, further justifying research on such solutions. A disadvantage of the materials under consideration is their inhomogeneous and stochastic structure—they are difficult to characterize and can lead to a lack of repeatability in the properties of the finished composite. Another area of research with a small number of publications is the use of epoxy resins, especially those used to make environmentally friendly composites. The goal of this study was to develop innovative, environmentally friendly composite materials and determine their abrasive properties based on a unique test setup. Due to the previously described drawbacks related to the stochastic nature of the reinforcement materials used, a series of standardized tests and observations were performed to determine the characteristics of the composite materials.

## 2. Experimental Methods

### 2.1. Composite Material

**Figure 1:**
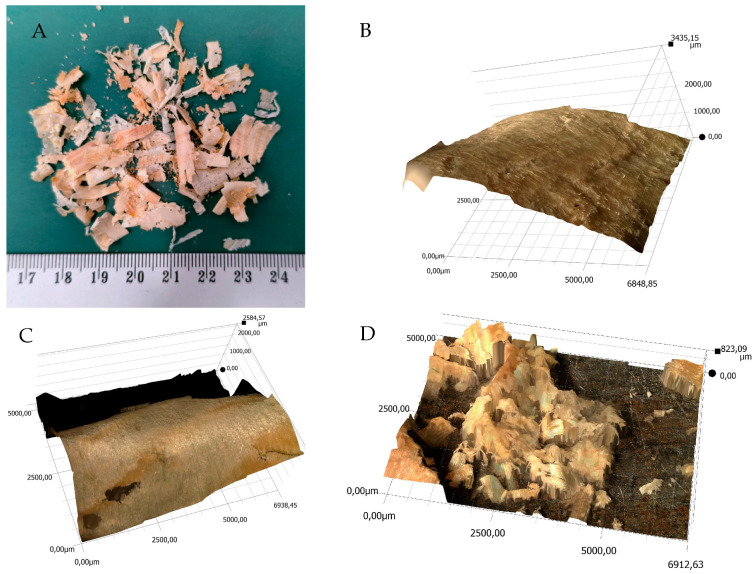
View of sample pine chips used to produce the samples; (**A**)—general view, (**B**)—optical 3D scan of a large chip fragment, (**C**)—optical 3D scan of a medium chip fragment, (**D**)—optical 3D scan of small chips and wood dust.

In order to prepare composites, natural materials were selected as reinforcement. The first of them was pine chips. The chips had irregular shapes; they were waste from pine wood processing. The material used to make the pine chips came from forests located in northern Poland. The chips were obtained during an industrial surface planning process; the processed material was pine wood kiln-dried to a relative humidity below 18%. The maximum planning depth was 3 mm. The chips—Figure 1A—were classified into three groups: large chips—Figure 1B, with a width exceeding 10 mm and a maximum thickness of approximately 0.6 mm; medium chips—Figure 1C, with a width exceeding 5 mm and a maximum thickness of approximately 0.2 mm; and fine chips and wood dust—Figure 1D. The lengths of the chips (large and medium) varied but did not exceed 15 mm. Collected chips were protected against contamination and moisture absorption.

Basalt flour used in experimental studies was a gray dust of natural origin. The chemical composition of the product, given in Table 1, and basalt flour fractions given in Table 2, were based on the producer’s (PPHU Dolpol) data sheet. The pH value was 9.0 ± 0.3. Basalt, used to make basalt flour, was obtained from southwestern Poland (Lower Silesia region).

The research presented in this thesis was conducted using EPODEX ECO epoxy resin and hardener, made by EPODEX GmbH, Krefeld, Germany, which, according to the manufacturer, are self-degassing. Additionally, resin is non-toxic and free of free bisphenol A. Product characteristics are given in Table 3.

Before starting the composite manufacturing process, molds were made and covered with an anti-adhesive layer to facilitate the removal of finished products from the mold. Work began with the mechanical mixing of resin with hardener in a 2:1 ratio, in accordance with the manufacturer’s recommendations.

Four types of samples were prepared with the composition shown in Table 4. The densities given in the resin and hardener data sheets obtained from the manufacturer were used to calculate the mass of the epoxy resin used. The resin density was 1.1295 g/cm^3^ while the hardener density was 1.05 g/cm^3^. After mixing in the appropriate proportions, the obtained resin density was 1.103 g/cm^3^. The proportions of the components were determined empirically based on material molding tests. The mass of the pine chips was selected so that, without the use of compression techniques for the reinforcement material, the chips filled the entire volume of the mold but did not protrude above the surface of the samples. The mass of the basalt was determined during the homogenization test so that the entire volume of the basalt flour was effectively wetted by the resin. The composite, consisting of resin, pine chips and basalt, was made from half the mass of the chips used to create the resin–pine chips composite and half the mass of the basalt flour used to create the resin–basalt flour samples. The mass of the resin was reduced to a minimum, allowing for the production of samples consistent with the description for the pine samples.

Based on the data presented in the table above, the percentage content (by weight) of natural materials in relation to the epoxy resin was determined. The results are presented in Table 5, rounded to the nearest whole number. The curing time required by the manufacturer is at least 16 h, and the time was extended to 60 days.

After appropriate hardening, the samples were cut using a hydroabrasive technique (waterjet cutter). In order to adapt the samples for strength tests, 3 standard samples were cut for the tensile test, 5 samples for the impact test and 6 cuboids for determining the density of the obtained composites.

### 2.2. Standardized and Optical Tests Measuring Equipment and Conditions

Due to the reinforcement materials used—pine chips, which are characterized by anisotropic structure, and basalt flour—and their stochastic distribution in the composite material, a series of standardized tests was performed to determine the mechanical properties of the tested composites. At least 5 measurement repetitions were used. The tests were performed under normal conditions.

The tensile test was performed on a Zwick Roell Z030, Ulm, Germany testing machine. The tensile speed was 1 mm/min. The test was aimed at determining Young’s modulus E, tensile strength σ and elongation/shortening ε (both for stretching and squeezing).

The impact test was performed on a Zwick Roell HIT 50P, Ulm, Germany impact test hammer. A 4 J hammer was used and the distance between the supports was 46 mm. The samples used were rectangular without a notch. The results of the impact test were used to determine the impact strength.

A Radwag MAC 110, Radom, Poland moisture analyzer was used to test the density from the mass and volumetric measurements. A total of 6 measurements were taken for each material. For the immersion method, the AXIS AD50, Gdańsk, Poland electronic scale was used (comparative method, for a smaller number of samples). A Sartorius Lab Instruments CPA225D-0CE, Göttingen, Germany laboratory scale was used to measure the mass losses of samples and counter-samples.

A Zwick hardness tester was used: Roell Shore D BH 04.3131.000, Ulm, Germany. Measurements were carried out on both the upper and lower surface of the samples. 

The macrostructure of the obtained composites was examined using a Keyence, Itasca, IL, USA, VHX-7000 Ver 1.4.17.3, System Ver 1.05 digital microscope with manufacturer’s software for image processing and measurements VHX Control System Ver 18.12.04.0A Ver 01.00.00.02.

### 2.3. Methodology and Technique of Abrasive Properties Determination

**Figure 2:**
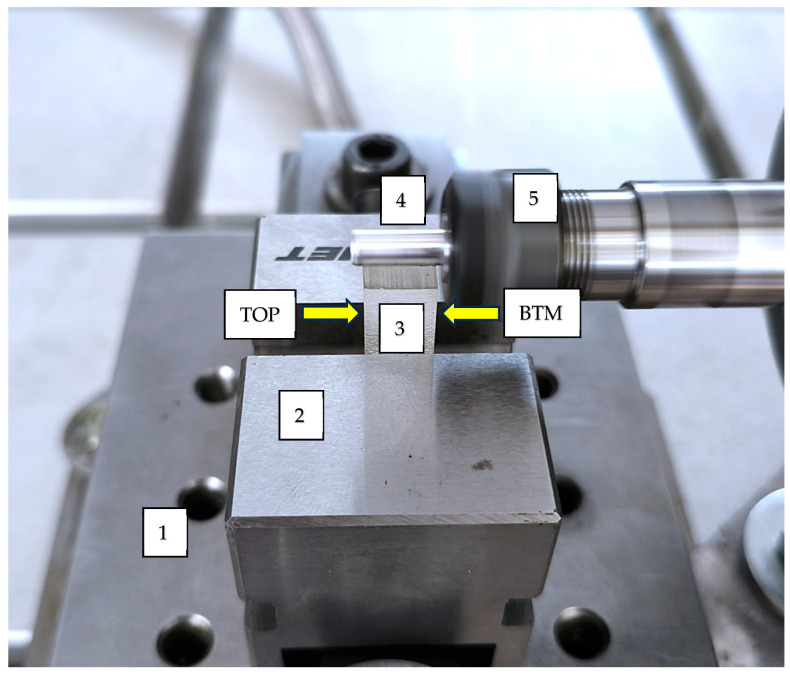
Stand zone for determining the abrasive properties of materials; 1—force gauge, 2—laboratory vice, 3—sample, 4—counter-sample, 5—electric spindle holder, sample’s orientation: TOP—top side, BTM—bottom side.

In order to determine the abrasive properties, an original test stand and methodology were developed. The solutions described in [24,25] were adapted. The test stand consisted of a guide equipped with a CNC drive controlled by the Linux CNC environment, on which an additional sliding guide with an electrospindle holder was mounted. The view of the sample mounting area and the counter-sample is shown in Figure 2.

A total of 10 samples of each composite material and the required number of counter-samples were prepared. The samples had a cuboid shape with transverse dimensions corresponding to the transverse dimensions of the samples used for strength tests and a length of 20–25 mm. The surfaces of the samples that were subjected to the friction test were milled to obtain the appropriate micro- and macro-geometric features. Before the experiment, the samples and counter-samples were weighed twice. The counter-samples were made by turning a 5 mm diameter shaft on a Doosan Lynx2100, Seoul, Republic of Korea, at a cutting speed v_c_ = 200 m/min and a longitudinal feed fv = 0.1 mm/rev. A Sandvik, Sandviken, Sweden, CoroTurn 107 SVHBL 2020K turning tool with a YG1, Songdo, Republic of Korea, VBMT 160404-UG cutting insert made of 3010 carbide was used. The length of the counter-sample was 25 mm. After the turning process, the Mahr, Göttingen, Germany, MarSurf GD120 profilometer was used to determine selected surface parameters (roughness parameters). The average values of the roughness parameters were Ra 0.77 μm, Rmax 4.1 μm and RSm 101 μm.

**Figure 3:**
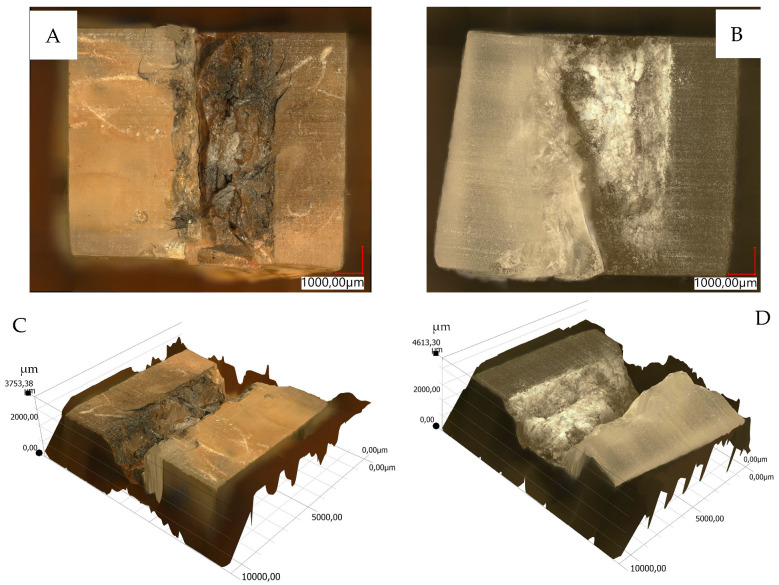
View of the samples used to perform the preliminary tests: (**A**,**C**)—sample RP, damaged in the 10th minute of the test, (**B**,**D**)—sample R, damaged in the 7th minute of the test.

The electrospindle was powered with a DC voltage of 12 V (nominal voltage) using a laboratory programmable stabilized power supply Korad KA3005P, Shenzen, China, with a maximum voltage of 30 V and a maximum current of 5 A, enabling operation in constant voltage mode with simultaneous current modulation. The power supply had the ability to read voltage and current in real time. The counter-sample, made of aluminum alloy EN AW-6082 (AlSi1MgMn, AlMgSi1 according to DIN, 3.2315 according to Werkstoff), was attached to the electrospindle using a collet. The samples were mounted in a laboratory vice attached to a Kistler, Winterthur, Switzerland, 9257B force gauge with a Kistler 5017 signal amplifier and a Kistler DAQ 5697 data acquisition system, along with DynoWare, version 2.6.5.16, force analysis software. The dynamometer and its accessories were used to determine and monitor the quasi-constant load force of the counter-sample pressing against the sample and to collect data for future tests. The number of spindle revolutions was determined using a Lutron, Taiwan, DT-1236L laser tachometer. The experiment consisted of two stages: the first stage defined the conditions for conducting the tests and the second was the main stage, where data acquisition was carried out. As a result of the first stage, it was determined that the voltage supplying the electrospindle would be equal to the nominal voltage. The (gravitational) force of the counter-sample pressing against the sample, adopted for further tests, was 9.45 N. The most important aspect of this stage of the tests was to determine the test time, which should be equal for each of the materials. Of all the materials used in the tests, the reference material, R, was destroyed the fastest, being damaged in the 7th minute of the test—Figure 3B,D. The RP material was damaged in the 10th minute of the test—Figure 3A,C.

The test time was set at 360 s (6 min). The current and the number of revolutions of the no-load electrospindle were measured, as well as the current and the number of revolutions in the 300th second of the test. After the tests, each sample (composite) and counter-sample (aluminum shaft) were weighed twice.

## 3. Results

### 3.1. Mechanical Properties Test Results

#### 3.1.1. Density

**Figure 4:**
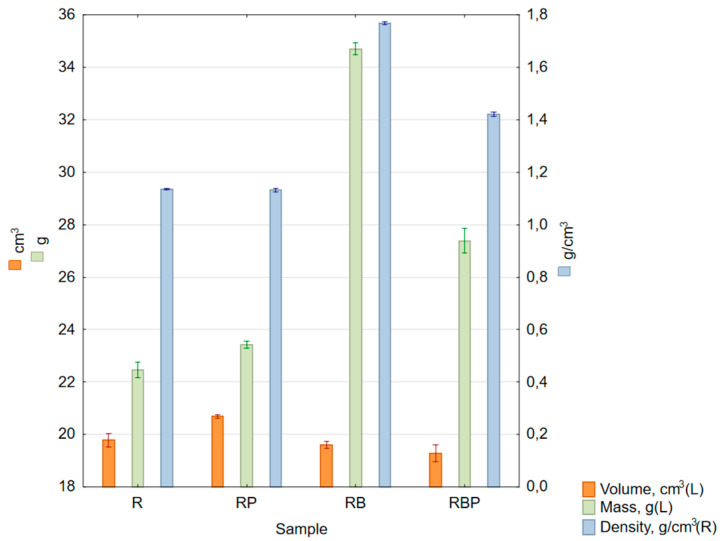
Box and whisker chart of mean values of sample volume (orange columns, left scale), mass (green columns, left scale) and density (blue columns, right scale); standard errors are indicated.

The results of the dry density measurements, i.e., based on the dimensions and mass of the samples, are presented in Table 6 and Figure 4. According to the Epodex data sheets, the density of the resin is 1.1295 g/cm^3^ and that of the hardener is 1.05 g/cm^3^. After mixing them in a 2:1 ratio, the density of the resin is 1.103 g/cm^3^.

Table 7 presents the results of immersion measurements performed for each third sample of each type of manufactured material.

#### 3.1.2. Tensile Strength

**Figure 5:**
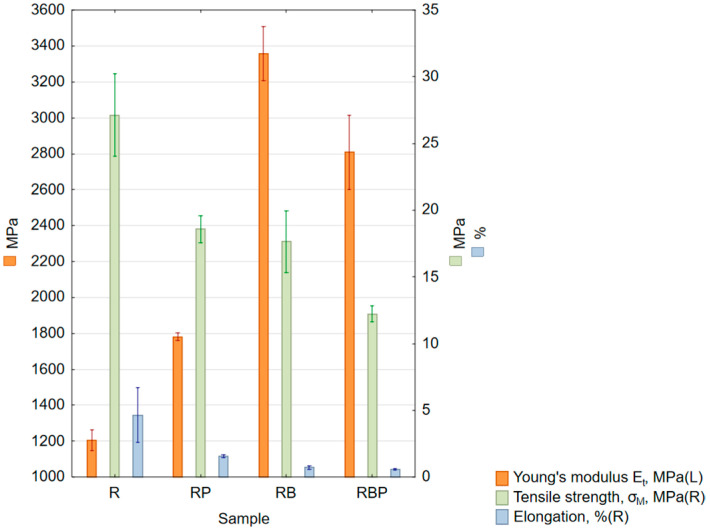
Box and whisker chart of mean values: Young’s modulus (orange columns, left scale), tensile strength (green columns, right scale) and elongation (blue columns, right scale); standard errors are indicated.

The results for samples subjected to the static tensile test are presented in Table 8 and Figure 5.

#### 3.1.3. Impact Strength

**Figure 6:**
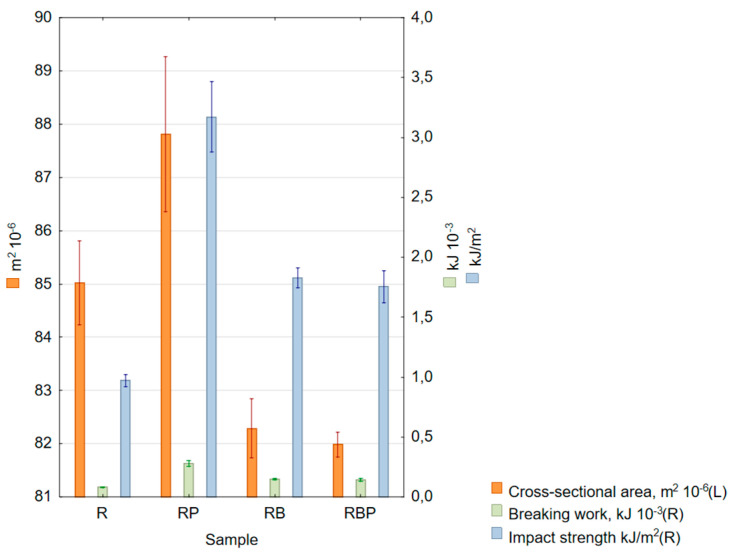
Box and whisker chart of mean values: cross-sectional area (orange columns, left scale), breaking work (green columns, right scale) and impact strength (blue columns, right scale); standard errors are indicated.

The measurement results of the impact test are presented in Table 9 and Figure 6.

#### 3.1.4. Compressive Strength

**Figure 7:**
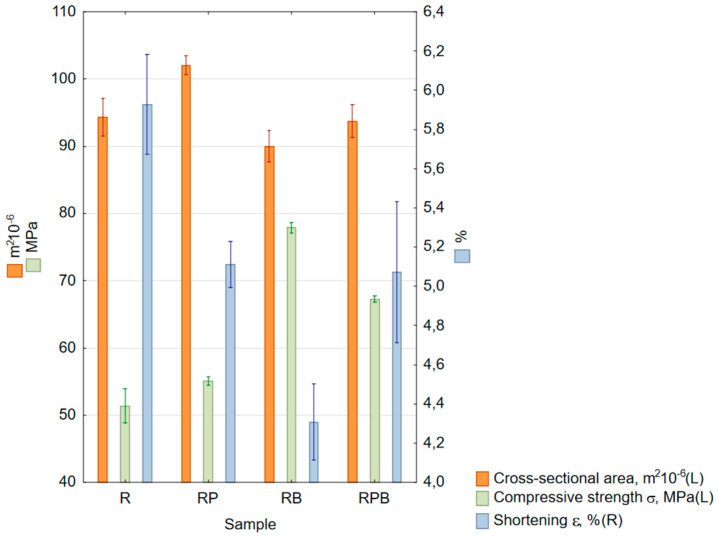
Box and whisker chart of mean values: cross-sectional area (orange columns, left scale), compressive strength (green columns, right scale) and shortening (blue columns, right scale); standard errors are indicated.

The compressive strength was also determined, where the maximum value of the compressive strength was assumed for the moment of loss of the ability to transfer an increasing load; this value was compared with the negative relative elongation of the sample—Table 10 and Figure 7.

#### 3.1.5. Hardness

**Figure 8:**
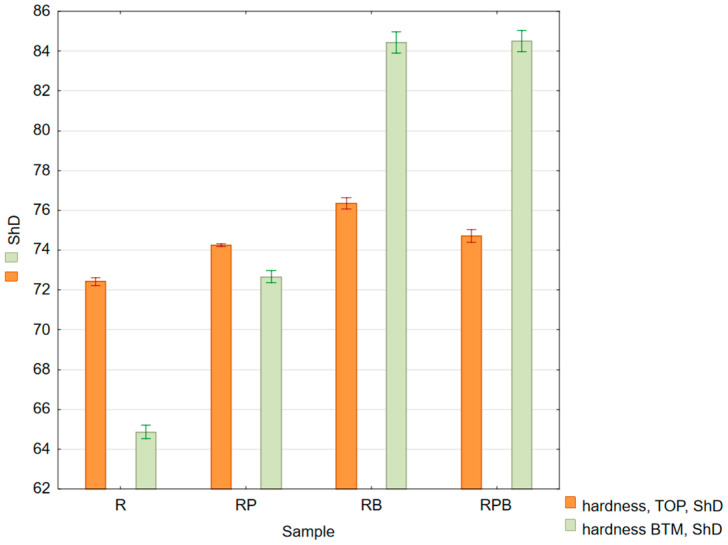
Box and whisker chart of mean hardness values: top side (orange columns), bottom side (green columns); standard errors are indicated.

The hardness test results are presented in Table 11 and Figure 8.

### 3.2. Macroscopic Structure and Surface Studies

**Figure 9:**
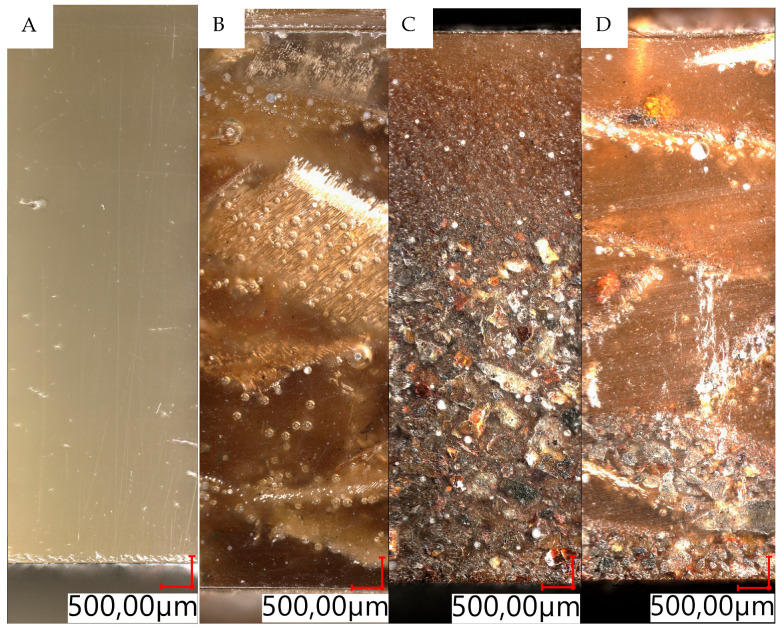
Composite structures comparison: (**A**)—resin, (**B**)—resin and pine chips, (**C**)—resin and basalt flour, (**D**)—resin, basalt flour and pine chips.

The next stage of the study was the observation of the structure of the obtained composites of natural origin using an optical microscope. For each of the prepared samples (one of each type), photos were taken in three different places on the sample surface. The comparison of the structures of the obtained composites and the control sample is presented in Figure 9.

**Figure 10:**
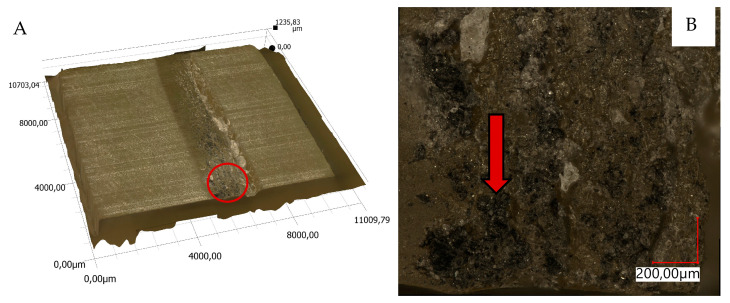
Three-dimensional view of the resin sample surface (R) after the abrasion test; (**A**)—location of the phenomena, (**B**)—indication of the phenomena.

**Figure 11:**
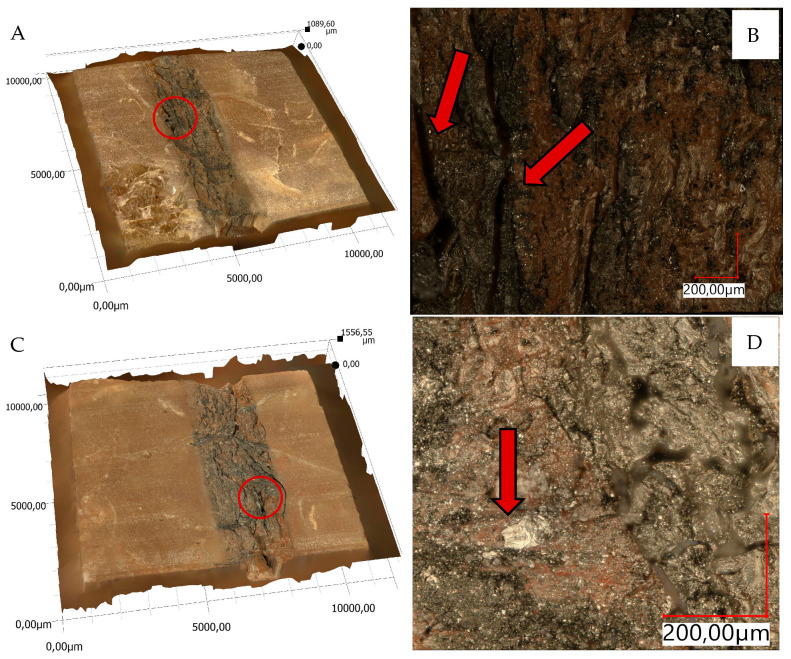
Three-dimensional view of the samples surfaces made of resin and pine chips (RP) after the abrasion test; (**A**,**C**)—location of the phenomena, (**B**,**D**)—indication of the phenomena.

**Figure 12:**
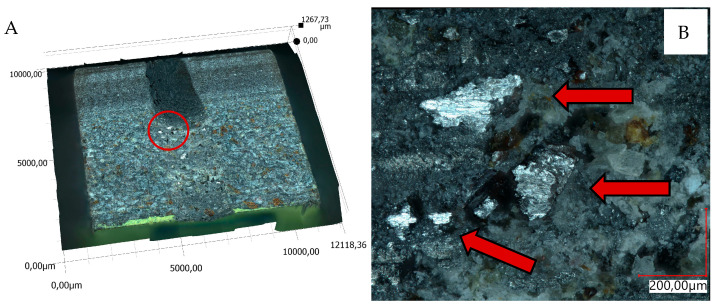
3D view of the surface of the resin and basalt (RB) sample after the abrasion test; (**A**)—location of the phenomena, (**B**)—indication of the phenomena.

**Figure 13:**
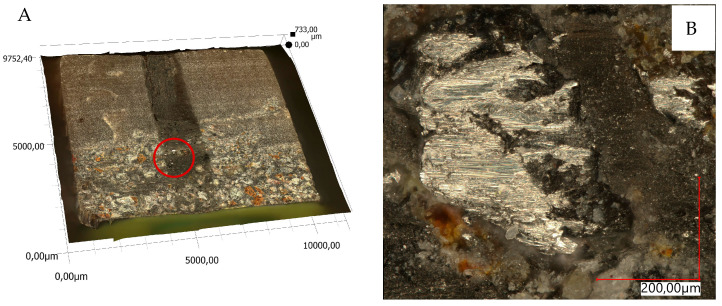
3D view of the sample surface made of resin, pine chips and basalt (RBP) after the abrasion test; (**A**)—location of the phenomena, (**B**)—indication of the phenomena.

**Figure 14:**
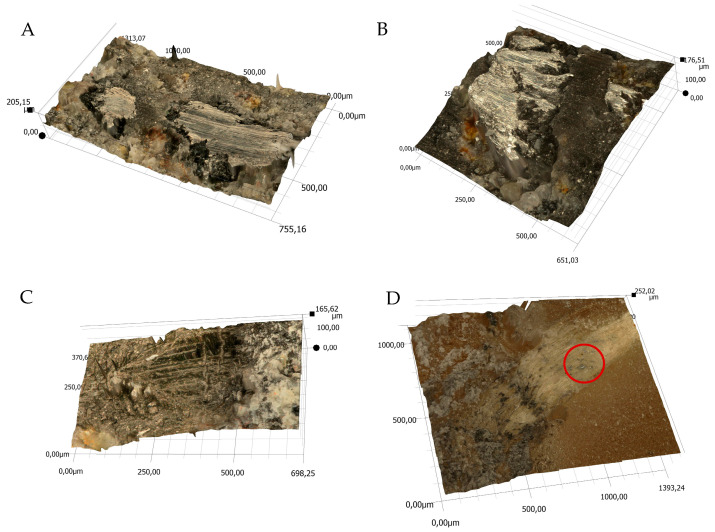
Three-dimensional view of the samples surface after abrasive properties test, scale expressed in µm: (**A**)—RB sample, (**B**)—RBP sample, (**C**)—RBP sample, (**D**)—RP sample; figure description in text.

The views of sample 3D surface scans are shown in Figure 10, Figure 11, Figure 12 and Figure 13. The figures on the left (parts A, C) show the location of the phenomena, indicated by the arrow, presented in the figures on the right (parts B, D). A 3D view of the sample surfaces after the abrasive properties test is shown in Figure 14.

### 3.3. Abrasive Properties Test Results

**Figure 15:**
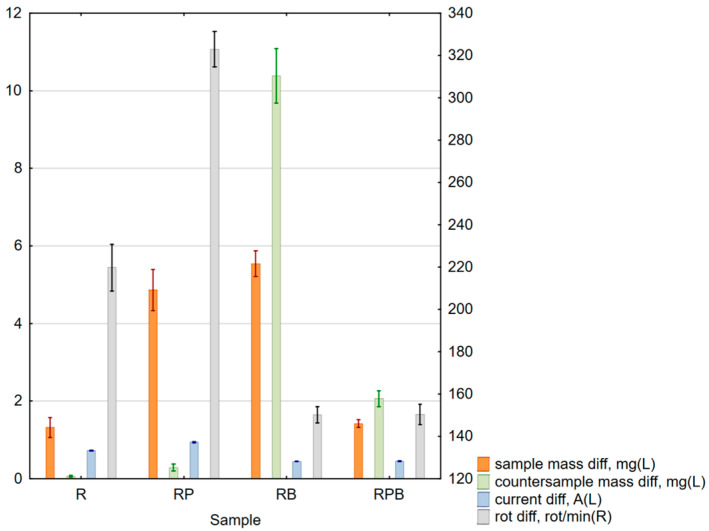
Box and whisker chart of average values: sample mass differential (orange boxes, left scale), counter-sample mass differential (green boxes, left scale), power current differential (blue boxes, left scale) and spindle rotation speed differential (gray baoxes, right scale); standard errors are indicated.

Abrasive properties test results are presented in Table 12 and Figure 15.

The averaged, maximum friction path, determined for the nominal value of the electrospindle rotational speed of 4253 rev/min from the following relationship:

S = ΠDnt/1000(1)

where

S—friction path, m;

D—counter-sample diameter, mm;

n—number of counter spindle revolutions, rev/min;

t—test time, min;

was 360 m, which means that, nominally, the counter-sample covered a friction distance of 1 m in 1 s, with an initial unit pressure of 1 N per 1 mm of contact length with the counter-sample.

The abrasion coefficient defined in [22] was determined from the following relationship:

W_z_ = m_c_/m_p_ (2)

where

W_z_—dimensionless abrasiveness index;

m_c_—counter-sample mass loss, mg;

m_p_—sample mass loss, mg.

The obtained average values of the W_z_ are presented in Table 13.

## 4. Discussion

### 4.1. Composite Materials Characteristic

#### 4.1.1. Density

No significant differences were observed in the density results obtained using both methods (from the mass and volumetric measurements and using the immersion method)—Table 6 and Table 7, Figure 4. Additionally, for the epoxy resin, the density value according to the manufacturer’s data was presented. The higher calculated value may result from measurements errors or differences in measured density values compared to the values declared by the resin manufacturer. Different density values for RP samples obtained using the weight–volume method and the immersion method indicate the presence of air bubbles, which was confirmed during macroscopic observations using a microscope.

#### 4.1.2. Tensile Strength

The lowest Young’s modulus value was obtained for the reference material (R)—epoxy resin—Table 8, Figure 5. This indicates that the natural materials used to make the composites fulfill their role as matrix reinforcement. A significant improvement in the stiffness of the obtained composites was achieved. The greatest increase was obtained using basalt flour (RB) and was 178% bigger than the reference material (R). A slightly lower result was obtained using a mixture of pine chips with basalt flour (RBP); the increase was 133%. For pine chips alone (RP), the Young’s modulus value increased (compared to the reference material—resin) by 47%. Pine chips increase the stiffness of the obtained composites to a lesser extent compared to basalt flour. In contrast to the Young’s modulus values, the tensile strength values decreased in each of the composites compared to the control samples. The largest decrease was obtained for the composite with a mixture of materials (RBP) and amounted to 55% compared to the value obtained for the epoxy resin (R). For pine chips (RP) and basalt flour (RB), the values obtained were similar and decreased by 32% and 35%, respectively. In work [16], concerning the tests of composites reinforced with wood dust, a decrease in the values of strength parameters during tensile tests, conducted at a speed of 1 m/min, was reported for the samples made with an increase in the content of wood filler wt.% of dust; a decrease of about 40% was observed for samples containing 5% of wood dust. A different result was demonstrated in study [18]. The mechanical strength of composites with a resin matrix reinforced with pine wood dust at 5, 15, 30, 40 and 50 wt.% was tested. A slight increase in tensile strength of 9.6% was observed for the pine wood dust content of 5 wt.%. The largest increase—by 44%—was noted for the 15% dust content, and the largest decrease—by 80.7%—was noted for the 50% dust content. Studies [26] on the effect of adding basalt flour to epoxy composites with basalt fiber reinforcement also found a reduction in tensile strength, especially with increasing basalt flour content. The maximum decrease, by approximately 30%, was observed for a 5% basalt flour content. In comparative studies [27] of the mechanical properties of resins reinforced with 30 wt.% basalt flour, synthetic resins with a density of 1.1–1.4 were used, with a maximum elongation of 4–7% and a tensile strength of 60–70 MPa. The actual density was 1.15 g/cm^3^, and the density with 30% basalt flour added was 1.324 g/cm^3^. The obtained tensile strength decrease was 12.69% and elongation decrease was 57%. Research [28] investigated the use of basalt fiber powder as reinforcement in combination with various polymer matrices. For basalt-reinforced synthetic resin with a resulting density of 1.31 g/cm^3^, 82.33 ShD, compared to the unreinforced resin with a density of 1.15 g/cm^3^, 81.21 ShD, the tensile strength decreased by 18.37%. For basalt-reinforced bio-based resin with a resulting density of 1.29 g/cm^3^, 83.52 ShD, compared to the unreinforced bio-based resin with a density of 1.14 g/cm^3^, 81.16 ShD, the tensile strength decreased by 23.75%. The lower tensile strength values obtained for composites with natural fillers may result from their inhomogeneous structure; agglomeration of reinforcement (especially pine chips or basalt particles deposited on pine chips) that can create weak points; variations in reinforcement distribution that can act as stress concentrators, the occurrence of defects in the structure (e.g., air bubbles), the distribution of reinforcement relative to the force direction; filler particles poorly bonded to the matrix; and the properties of the reinforcement materials (basalt being brittle, wood being anisotropic). Moreover, the RBP composite has a highly anisotropic structure in which the basalt particles sediment at the bottom. The highest elongation value was obtained for pure resin and its average was 4.6%. Adding reinforcement in the form of natural materials stiffened the produced composites; therefore, the elongation values decreased. A reduction in elongation of approximately 13% (from 3.8% to 3.3%) was also observed during tensile testing for samples containing 5% flour [26]. An increase in stiffness was determined in each case. A maximum increase of 21% was achieved for a 2.5% basalt flour content relative to the resin alone. The obtained research results, both from other authors and our own research, indicate that basalt flour has a greater effect on the ability to deform than pine chips, which is consistent with its mechanical properties as a hard and stiff filler.

#### 4.1.3. Impact Strength

The obtained results—Table 9, Figure 6—of impact tests show that, for each of the obtained composites, adding natural material increased the impact strength values. The highest value, which increased impact strength by 227% compared to pure epoxy resin, was obtained for epoxy composites with pine chips—on average, 3.172 kJ/m^2^. The largest scatter from the measured mean was also observed here, which is probably related to the stochastic arrangement of chips in the sample. The increase in impact strength values for composites with basalt flour and a mixture of flour with pine chips was 88% and 80%, respectively. In work [26], concerning hybrid composites made of basalt fibers and basalt flour, a reduction in impact strength of approximately 16% was observed for a 2.5% basalt flour content. The difference in the obtained results may result from different material structures—in the case of hybrid composites, the addition of basalt flour resulted in a reduction in the values of all tested mechanical properties due to the introduction of dispersed reinforcement into the composite material, which caused the formation of agglomerates (defects) in the epoxy matrix.

#### 4.1.4. Compressive Strength

The highest compressive strength of 76.41 MPa was recorded during the RB composite compression test, and the lowest of 51.36 MPa was recorded for the R reference material—Table 10, Figure 7. The increase in compressive strength in this case was 51.67%. Strengthening the structure in each case resulted in an increase in compressive strength—for the RP material, it was 55.11 MPa (+7.3%), and for RBP, 65.61 MPa (+29.03%), which clearly indicates a significant share of basalt compressive strength in the overall compressive strength of the composite material. The obtained compressive strength values occurred for very similar values of sample shortening and, for the RP and RBP samples, the differences compared to the R reference material did not exceed 10%. In the case of the RB sample, an about 20% reduction in the sample shortening value was noted, which was caused by a significant content of brittle material in the volume of the composite. In study [22] concerning the use of fly ash, basalt flour and quartz sand in various proportions, it was shown that the addition of basalt resulted in an increase in compressive strength by 106%. The highest increase in drainage strength was observed for 50 wt.% of basalt flour content in the total mass of the composite material reinforcement. The authors of [29] prepared composites from polyester resin, characterized by a tensile strength value of 55 MPa and elongation of 6.5%, and basalt powder with varying grain sizes and percentages. Compression test results were performed and presented for various basalt wt.% contents and granulations. The minimum compression strength value of 105 MPa was obtained for a basalt content of 60%, and the maximum value of 155 MPa was obtained for a basalt content of 30%. Different fractions of basalt grain sizes (granulation from 50 to 1000 μm) to those in the current work were used.

#### 4.1.5. Hardness

The greatest difference in the obtained hardness measurement results in relation to the reference material R was observed for the RP material, both for the upper and lower surfaces—Table 11 and Figure 8. The increase in hardness on the upper surface of the samples in all analyzed cases was small and amounted to a maximum of 5.5% for the RB sample. The increase in the hardness value on the lower surface of the samples was greater and reached a maximum of about 30% for the RB and RBP samples. The obtained results indicate that sedimenting basalt was the factor most strongly shaping the tested feature on the lower surface of the composite.

### 4.2. Material’s Structure

The structure of the epoxy resin in the reference material is uniform—Figure 9. It can be seen that the resin, according to the manufacturer’s descriptions, degasses itself. Only a few small air bubbles were observed—Figure 9A. Observations of the composite of epoxy resin and pine chips (RP)—Figure 9B—indicate a multidirectional arrangement of wood fibers. They are arranged in bands of different densities. The chips have irregular shapes of small thickness, which facilitate their mechanical bonding with the resin, improving adhesion. The arrangement of pine chips was stochastic, which reduces anisotropy. Numerous air bubbles of different diameters can be observed in the epoxy resin. They are irregularly arranged, but a particularly high density is observed in pine chip surfaces. This may suggest that they were blocked by them and that there was no possibility of self-deaeration. Another reason could be interaction with wood fibers. Too fast a resin curing process, which could cause bubbles to be closed in the composite structure, can be ruled out, because the curing time for all composites was the same and long (several hours). The largest bubble observed was 281 µm. On average, they were about 100 µm. Observations of the epoxy resin–basalt flour (RB) composite—Figure 9C—showed the occurrence of irregular, sharp grains of various sizes, mostly dark brown in color. There were also red and dark yellow inclusions, which are not visible to the naked eye. Basalt sedimentation and a change in sample density were observed. The lower part shows a higher concentration of basalt grains than the upper part. The flour fell to the bottom of the mold in a shorter time than the time needed for the resin to completely cure. Similarly to the composite with pine chips (RP), small round dots were found, which are air bubbles. Their number is much lower and they occur in only a few places. It can be seen that, near the larger grains of basalt flour, the bubbles are also larger and reach a size of up to 130 µm. In the upper part, they are much smaller and their size oscillates around 50 µm. In the case of epoxy resin reinforced with two natural materials (RBP)—Figure 9C—similarly to earlier, basalt flour, due to its density, sank to the bottom of the mold. A small part of the flour grains stopped on the pine chips. In the lower part of the samples, in addition to basalt flour grains, there are also pine chips. The number of air bubbles is comparable to the amount in the composite with basalt flour alone; however, similarly to the composite with chips (RP), the density of bubbles is higher near the chips, especially those arranged horizontally.

### 4.3. Abrasive Properties

The obtained results of sample and counter-sample mass losses (Table 12, Figure 15) during the developed test for determining abrasive properties clearly indicate the usefulness of the proposed measurement technique; however, their interpretation is difficult due to the numerous phenomena occurring during the test.

The measured and determined quasi-constant force abrasiveness test values of composite materials were statistically analyzed using the Statistica version 13 program and are presented in Table 14. The analysis showed the validity of all tested cases.

**Figure 16:**
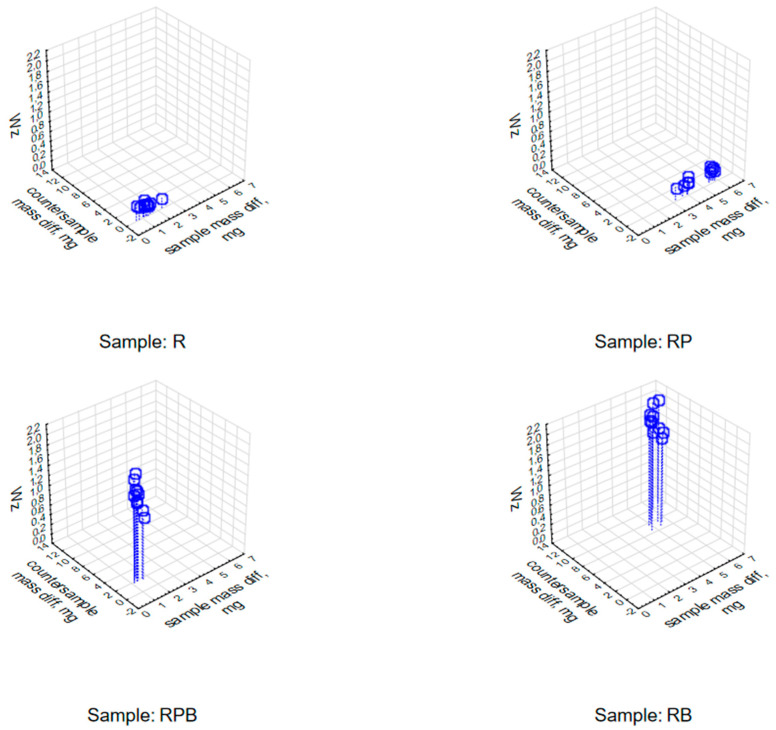
Categorized graph of changes in the W_z_ index value depending on the mass loss of the sample and counter-sample.

The analysis of the changes in the value of the W_z_ index showed that it reached diverse and repeatable values depending on the loss of sample and counter-sample mass. Scatter diagrams—Figure 16—illustrate the relationship between the loss of sample and counter-sample mass and the value of the W_z_ index for individual pairs of materials subjected to the abrasiveness test.

**Figure 17:**
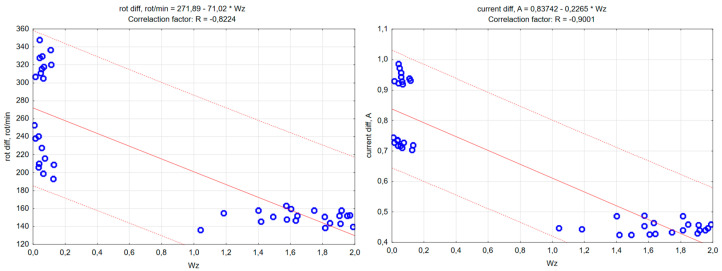
Scatter plots (description in the text).

The next stage was to determine the relationship between the abrasiveness index values and the values of changes in the rotational speed and supply current of the electrospindle during the tests. The analyses performed indicate a strong correlation—at the level of 0.9—between the abrasiveness index values determined during the analyses, the changes in the value of the current supplying the electrospindle and the changes in the rotational speed of the electrospindle measured during the experiment. The obtained analysis results are presented in graphic form—scatter graphs of the values of changes in the number of electrospindle revolutions (on the left) and changes in the value of the electrospindle supply current (on the right) depending on the value of the W_z_. index—Figure 17. The figure indicates the range of regression, corresponding to the 95% confidence level, the regression red line and the form of the regression function, with a red dashed line.

**Figure 18:**
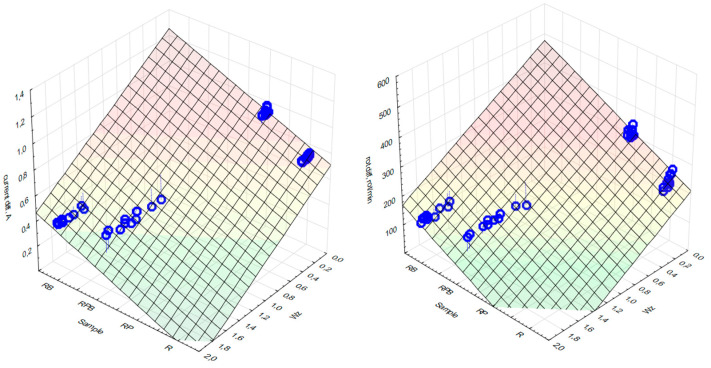
Changes in the power current value (**left**) and rotational speed (**right**) of the electrospindle for individual composite materials and different values of the W_z_ index.

A regression analysis of the changes in the current value and the number of revolutions of the electrospindle depending on the loss of mass of both the sample and the counter-sample was performed. The results are presented in Table 15 and Table 16. Statistically significant values are marked in red. High values of the correlation coefficients indicate high sensitivity of the measurements of changes in the current and the number of revolutions, while the effective determination of the abrasive properties of the material must include the analysis of both values simultaneously—Figure 18.

Material surface observations showed significantly different surfaces after the test depending on the material. The reference material (R) was characterized by the occurrence of numerous damages in the form of tear-outs and material discontinuities in the layers in contact with the counter-sample. Samples containing pine chips (RP) were characterized by the occurrence of numerous burns and discolorations as well as single tear-outs. The three-component material (RBP) turned out to be a material with the form of abrasive wear characterized by a lack of damage in the zone of contact with the counter-sample. The RB composite showed similar features. Observations of the sample surfaces after testing the abrasive properties of the composites revealed similarities to phenomena occurring during abrasive machining. Figure 14A,B show basalt grains protruding above the composite matrix that exhibit abrasive grain characteristics. In abrasive machining, the thickness of the cut layer is relatively small compared to the radius of curvature of the active abrasive grain tip. This is caused by large negative rake angle values, for which a relatively small volume of material is removed as chips while a larger volume is plastically deformed material. The amount of plastically deformed material can be 75 times greater than the volume of removed chips. In such a case, a large portion of the thermal energy generated by the processes is transferred to the workpiece [30], as confirmed by Figure 10B, Figure 11B and Figure 14C, which indicate the presence of intensely oxidized areas surrounded by areas where matrix and reinforcement material have been removed. Observations of the sample surfaces revealed adhesion of the counter-sample material to the basalt grains—Figure 12B, Figure 13B and Figure 14A,B—indicating that the counter-sample material exceeded the energy required for its plasticization. The products resulting from the microdestruction processes are very small particles of material separated by decohesion, ranging in size from a few to a dozen or so μm. The observations and analyses performed indicate the possibility of adapting Shaw’s model [31] to determine the mechanism of counter-sample material removal during the abrasiveness test. The occurrence of a few metallic particles of very small sizes (a few μm) on the surfaces of the RP samples is intriguing—Figure 11C,D and Figure 14D. This may be the result of contamination of the sample material with single basalt grains, which resulted in the formation of aluminum chips, or contamination with the chips themselves.

Based on the experiments and analysis of our own research results and those included in the available literature, the following research directions were identified:

- Investigating the properties of composite materials reinforced with basalt powder with varying wt.% of basalt and pine chips (varying the pine chip–basalt powder–resin) relative to the matrix material, attempting to compress pine chips to increase their volumetric share in the composite,
- Performing an energy analysis (including the composite’s thermal conductivity and heat capacity determination) of the counter-sample friction process, thermal imaging and thermal measurements analysis and kinematic pair interactions (e.g., friction force measurements), based on which it will be possible to formulate a model of the abrasive behavior in basalt-particle-reinforced resin composites.

## 5. Conclusions

Based on the analysis of available scientific reports and the original research conducted, the following conclusions were formulated:

- The presented method for determining the abrasive properties of composite materials using a quasi-constant-force friction test of a counter-sample against the sample surface is significant and adequate for the diverse characteristics of the tested materials. Changes in the Wz index value were good indicators of the abrasive properties of the composite materials. Changes in the supply current and rotational speed of the electrospindle motor may also be useful for determining the abrasive properties of composite materials; however, they require further work and analysis, particularly in terms of the energy indices associated with the counter-sample–sample friction process.
- The material with the highest abrasiveness was a composite with a resin matrix and basalt reinforcement (RB), for which the abrasiveness index (Wz) was 1.873. The composite with the lowest abrasiveness was epoxy resin reinforced with pine chips (RP), with a Wz index of 0.059. Despite the significant difference in the percentage of basalt (RB—58 wt.%, RBP—35 wt.%), the RB and RPB composites were characterized by high and similar abrasion index values (the Wz value for RBP was 1.456). The analyses conducted clearly indicate that basalt grains embedded in the polymer matrix possess abrasive properties, enabling decohesion of the counter-sample material due to plasticization of the counter-sample material. The tensile strength decreased in each of the composites, indicating the influence of the materials used, particularly the basalt flour, on the increased brittleness of the resulting materials and limited plastic deformation capacity. The obtained results may have been influenced by the structural characteristics of the composites made from natural materials, such as agglomeration of reinforcement, the occurrence of defects in the structure (e.g., air bubbles) or variations in reinforcement distribution, which could result in the occurrence of weak points or stress concentrations.
- The greatest reduction in tensile strength—by 55%—was obtained for the composite with pine chips and basalt (12.24 MPa for RBP, in comparison to 27.14 MPa for R). The Young’s modulus of the resulting composites increased compared to the epoxy resin (reference samples). This indicates that the natural additives used increased the stiffness of the resulting composites. The increase after adding basalt flour was 178% (3358.31 MPa for RB, in comparison to 1205.53 MPa for R) and, after adding pine chips, it was 47% (1780.36 MPa for RBP, in comparison to 1205.53 MPa for R). The multicomponent composite (RBP) was characterized by a Young’s modulus of 2809.1 MPa, representing a 133% increase compared to the resin alone.
- The use of reinforcing materials in the form of pine chips and basalt flour to produce environmentally friendly composites resulted in increased compressive strength values compared to the matrix material. The largest increase—by 51.67%—was recorded for RB samples (76.41 for RB, in comparison to 51.67 for R), and the smallest—by 7.3%—was recorded for RP samples (55.11 for RP in comparison to 51.36 for R). The obtained results indicate that basalt, as a material with high hardness and a significant wt.% share, was the most significant factor influencing the final compressive strength. Basalt flour, dispersed in the composite structure, can contribute to stress dissipation, which allows for the delay of plastic deformation appearance, which can increase the overall strength of the material.

## Figures and Tables

**Table 1:** Chemical composition of basalt particles.

Name of Chemical Component	Percentage Content, wt.%
silica (SiO_2_)	40–50%
alumina (Al_2_O_3_)	0–20%
iron oxide (Fe_2_O_3_)	5–10%
magnesium (MgO)	5–15%
calcium (CaO)	5–15%
other minerals	5%

**Table 2:** Basalt flour fractions.

Grain Size	Percentage Content, wt.%
Fraction content > 0.5 mm	12.8 ± 0.9
Fraction content 0.1–0.5 mm	55.3 ± 3.9
Fraction content 0.06–0.1 mm	7.49 ± 0.5
Fraction content < 0.06 mm	24.5 ± 1.7

**Table 3:** Epoxy resin and hardener properties.

Characteristic	Resin	Hardener
Consistency	Fluid	Fluid
Color	Light yellow	Yellowish
Smelling	Characteristic	Amin
Initial boiling point and boiling range	201 °C	-
Freezing point	−5 °C	-
Flash point	140 °C	101 °C
Ignition temperature	>200 °C	380 °C
Autoignition temperature	Product does not spontaneously ignite	Product does not spontaneously ignite
Explosive properties	Product is not explosive	Product is not explosive
Steam pressure at 20 °C	<2 hPa	0.1 hPa
Density at 20 °C	1.1295 g/cm^3^	1.05 g/cm^3^
Viscosity: dynamic at 25 °C	-	120–240 mPas

**Table 4:** Quantitative composition of the components used to produce the samples.

Sample	Epoxy Resin	Basalt Flour	Pine Chips
R; Resin	330.9 g (300 mL)	-	-
RP; Resin, pine	330.9 g (300 mL)	-	12 g
RB; Resin, basalt	220.6 g (200 mL)	300 g	-
RBP; Resin, basalt, pine	275.75 g (250 mL)	150 g	6 g

**Table 5:** Content of composite components.

Composite	Ratio Value (in wt.%)
RP resin–pine	97:3
RB resin–basalt	42:58
RBP resin–basalt–pine	64:35:1

**Table 6:** Volume, mass and density of samples; average value (top—no mark), minimum and maximum values.

	R; Resin	RP; Resin, Pine	RB; Resin, Basalt	RBP; Resin, Basalt, Pine
Volume, cm^3^	19.79 min 19.07 max 20.61	20.69 min 20.42 max 20.89	19.36 min 19.17 max 20.07	19.28 min 18.23 max 20.08
Mass, g	22.46 min 21.58 max 23.34	23.42 min 22.97 max 23.82	34.7 min 33.81 max 35.31	27.39 min 26.02 max 29.21
Density, g/cm^3^	1.14 min 1.13 max 1.15	1.23 min 1.1 max 1.15	1.77 min 1.76 max 1.79	1.42 min 1.4 max 1.45

**Table 7:** Results of the verification density measurement using the immersion method.

Sample Material (Each 3rd Sample)	Density, g/cm^3^
R_3_	1.144
RP_3_	1.137
RB_3_	1.784
RBP_3_	1.483

**Table 8:** Tensile test results: average value (top—no mark), minimum and maximum values.

	R; Resin	RP; Resin, Pine	RB; Resin, Basalt	RBP; Resin, Basalt, Pine
Young’s modulus E_t_, MPa	1205.53 min 1147.71 max 1263.36	1780.36 min 1744.57 max 1819.39	3358.31 min 3187.84 max 3656.7	2809.1 min 2530.1 max 3212.34
Tensile strength σ_M_, MPa	27.14 min 24.06 max 30.21	18.57 min 16.59 max 20.04	17.64 min. 14.22 max 22.11	12.24 min 11.12 max 13.07
Elongation, %	4.65 min 2.61 max 6.69	1.56 min 1.33 max 1.74	0.71 min 0.49 max 0.99	0.59 min 0.53 max 0.7

**Table 9:** Impact test results: average value (top—no mark), minimum and maximum values.

	R; Resin	RP; Resin, Pine	RB; Resin, Basalt	RBP; Resin, Basalt, Pine
Cross-sectional area, m^2^ 10^−6^	85.02 min 84.23 max 85.81	87.81 min 83.61 max 92.63	82.29 min 81.15 max 84.35	81.99 min 81.39 max 82.49
Breaking work kJ 10^−3^	0.08 min 0.08 max 0.09	0.28 min 0.22 max 0.34	0.15 min 0.13 max 0.17	0.14 min 0.12 max 0.17
Impact strength kJ/m^2^	0.97 min 0.92 max 1.02	3.17 min 2.46 max 3.91	1.83 min 1.58 max 2.04	1.76 min 1.47 max 2.04

**Table 10:** Uniaxial compression test results: average value (top—no mark), minimum and maximum values.

	R; Resin	RP; Resin, Pine	RB; Resin, Basalt	RBP; Resin, Basalt, Pine
Cross-sectional area, m^2^ 10^−6^	94.29 min 85.75 max 101.85	102.03 min 98.66 max 106.72	89.99 min 83.19 max 95.1	95.75 min 85.09 max 98.59
Compressive strength σ, MPa	51.36 min 44.08 max 58.61	55.11 min 53.49 max 57.15	77.9 min 76.41 max 80.94	66.27 min 65.61 max 68.49
Shortening ε, %	5.93 min 5.36 max 6.85	5.11 min 4.77 max 5.33	4.31 min 4.00 max 5.01	5.07 min 4.33 max 6.38

**Table 11:** Hardness test results: average value (top—no mark), minimum and maximum values.

	R; Resin	RP; Resin, Pine	RB; Resin, Basalt	RBP; Resin, Basalt, Pine
Hardness TOP, ShD	72.4 min 71.9 max 72.9	74.2 min 74.1 max 74.5	76.4 min 75.4 max. 77.0	74.7 min 73.9 max 75.6
Hardness BTM, ShD	64.9 min 64.2 max 66.1	72.7 min 71.6 max 73.5	84.4 min 82.9 max 85.8	84.5 min 83.3 max 86.2

**Table 12:** Measurement results of the counter-sample and sample mass loss, power current and spindle speed changes: average values (top—no mark), minimum and maximum values.

	R; Resin	RP; Resin, Pine	RB; Resin, Basalt	RBP; Resin, Basalt, Pine
Counter-sample mass loss, mg	0.062 min 0.02 max 0.145	0.288 min 0.09 max 0.615	10.385 min 8.58 max 12.845	2.066 min 1.73 max 2.785
Sample mass loss, mg	1.32 min 0.65 max 2.23	4.866 min 3.23 max 5.81	5.544 min 4.73 max 6.73	1.419 min 1.1 max 1.67
Current change, A	0.7262 min 0.705 max 0.746	0.9404 min 0.92 max 0.986	0.444 min 0.434 max 0.458	0.4548 min 0.425 max 0.487
Revs change, rev/min	219.8 min 193 max 253	323 min 305 max 348	150.2 min 139 max 158	150.4 min 136 max 163

**Table 13:** Values of the W_z_ for each type of sample: average values (top—no marking) and minimum and maximum.

	R; Resin	RP; Resin, Pine	RB; Resin, Basalt	RBP; Resin, Basalt, Pine
W_z_	0.047 min 0.009 max 0.129	0.059 min 0.015 max 0.109	1.873 min 1.745 max 1.970	1.456 min 1.039 max 1.844

**Table 14:** Statistical analyses of measured and determined quantities during abrasiveness test.

	Avg	Conf.Int. −95%	Conf.Int. +95%	Min	Max	Var	Std. Dev.	Std Error
sample mass diff, mg	3.21	2.56	3.86	0.65	6.73	4,13	2.032	0.321
counter-sample mass diff, mg	3.13	1.78	4.48	0.02	12.84	17.77	4.216	0.666
current diff, A	0.64	0.57	0.70	0.42	0.98	0.04	0.210	0.033
rot diff, rot/min	210.47	187.35	233.59	136	348	5226.25	72.29	11.430
W_z_	0.86	0.59	1.13	0.009	1.98	0.701	0.837	0.132

**Table 15:** Summary of regression analysis of the current diff, A relative to the counter-sample mass diff, mg and sample mass diff, mg variables.

	Dependent Variable Regression Summary Current Diff, A R= 0.91921496 R^2^ = 0.84495614, Corrected R^2^ = 0.83657539 F(2.37) = 100.82 *p* < 0.00000, Standard Error (SE) of Estimation: 0.08513					
	β	SE β	b	SE b	t(24)	*p*
free parameter			0.553301	0.025667	21.5571	0.00
sample mass diff, mg	0.80352	0.081221	0.083239	0.008414	9.8930	0.00
counter-sample mass diff, mg	−1.14473	0.081221	−0.057176	0.004057	−14.0940	0.00

**Table 16:** Summary of regression analysis of the rot diff, rot/min relative to the counter-sample mass diff, mg and sample mass diff, mg variables.

	Dependent Variable Regression Summary: Rot Diff, Rot/Min R= 0.94125460 R^2^ = 0.88596022, Corrected R^2^ =0.87979591 F(2.37)= 143.72 *p* < 0.00000, Standard Error (SE) of Estimation: 25.064					
	β	SE β	b	SE b	t(24)	*p*
free parameter			167.4566	7.556617	22.1603	0.00
sample mass diff, mg	0.91595	0.069658	32.5727	2.477148	13.1493	0.00
counter-sample mass diff, mg	−1.14739	0.069658	−19.6733	1.194358	−16.4718	0.00

## Data Availability

The original contributions presented in this study are included in the article. Further inquiries can be directed to the corresponding author.
